# (3*E*,5*E*)-1-Acryloyl-3,5-bis­(2,4-dichloro­benzyl­idene)piperidin-4-one hemihydrate

**DOI:** 10.1107/S1600536811016023

**Published:** 2011-05-07

**Authors:** Alireza Basiri, Vikneswaran Murugaiyah, Hasnah Osman, Madhukar Hemamalini, Hoong-Kun Fun

**Affiliations:** a School of Pharmaceutical Sciences, Universiti Sains Malaysia, 11800 USM, Penang, Malaysia; b School of Chemical Sciences, Universiti Sains Malaysia, 11800 USM, Penang, Malaysia; c X-ray Crystallography Unit, School of Physics, Universiti Sains Malaysia, 11800 USM, Penang, Malaysia

## Abstract

The asymmetric unit of the title compound, C_22_H_15_Cl_4_NO_2_·0.5H_2_O, consists of a (3*E*,5*E*)-1-acryloyl-3,5-bis­(2,4-dichloro­benzyl­idene)piperidin-4-one mol­ecule and a half-mol­ecule of water (the O atom of the water mol­ecule lies on a twofold axis). The piperidin-4-one ring adopts an envelope conformation. The dihedral angle between the two terminal benzene rings is 8.84 (11)°. In the crystal, mol­ecules are connected by C—H⋯O hydrogen bonds forming supra­molecular chains along the *c* axis. Furthermore, adjacent chains are inter­connected by the water mol­ecules *via* O—H⋯O hydrogen bonds.

## Related literature

For details and applications of α,β-unsaturated carbonyl compounds, see: Oh *et al.* (2006 ▶); El-Subbagh *et al.* (2000 ▶); Husain *et al.* (2006 ▶); Favier *et al.* (2005 ▶). For details of the preparation, see: Dimmock *et al.* (2000 ▶). For ring conformations, see: Cremer & Pople (1975 ▶).
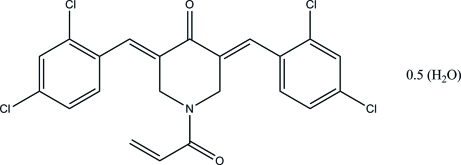


## Experimental

### 

#### Crystal data

2C_22_H_15_Cl_4_NO_2_·H_2_O
*M* *_r_* = 952.32
Monoclinic, 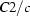

*a* = 27.0296 (12) Å
*b* = 11.3031 (5) Å
*c* = 18.9580 (14) Å
β = 133.807 (2)°
*V* = 4180.0 (4) Å^3^
*Z* = 4
Mo *K*α radiation
μ = 0.59 mm^−1^
*T* = 296 K
0.41 × 0.22 × 0.09 mm

#### Data collection

Bruker SMART APEXII CCD area-detector diffractometer
Absorption correction: multi-scan (*SADABS*; Bruker, 2009 ▶) *T*_min_ = 0.794, *T*_max_ = 0.947
22264 measured reflections
6084 independent reflections
3314 reflections with *I* > 2σ(*I*)
*R*_int_ = 0.035

#### Refinement

*R*[*F* ^2^ > 2σ(*F* ^2^)] = 0.050
*wR*(*F* ^2^) = 0.140
*S* = 1.04
6084 reflections
273 parameters
H atoms treated by a mixture of independent and constrained refinement
Δρ_max_ = 0.32 e Å^−3^
Δρ_min_ = −0.30 e Å^−3^

### 

Data collection: *APEX2* (Bruker, 2009 ▶); cell refinement: *SAINT* (Bruker, 2009 ▶); data reduction: *SAINT*; program(s) used to solve structure: *SHELXTL* (Sheldrick, 2008 ▶); program(s) used to refine structure: *SHELXTL*; molecular graphics: *SHELXTL*; software used to prepare material for publication: *SHELXTL* and *PLATON* (Spek, 2009 ▶).

## Supplementary Material

Crystal structure: contains datablocks global, I. DOI: 10.1107/S1600536811016023/sj5130sup1.cif

Structure factors: contains datablocks I. DOI: 10.1107/S1600536811016023/sj5130Isup2.hkl

Supplementary material file. DOI: 10.1107/S1600536811016023/sj5130Isup3.cml

Additional supplementary materials:  crystallographic information; 3D view; checkCIF report

## Figures and Tables

**Table 1:** Hydrogen-bond geometry (Å, °)

*D*—H⋯*A*	*D*—H	H⋯*A*	*D*⋯*A*	*D*—H⋯*A*
O1*W*—H1*W*1⋯O2^i^	1.05	2.19	3.180 (3)	157
C4—H4*A*⋯O1^ii^	0.93	2.29	3.186 (3)	162

Symmetry codes: (i) 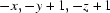; (ii) 
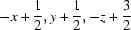
.

**Fig. 1.**
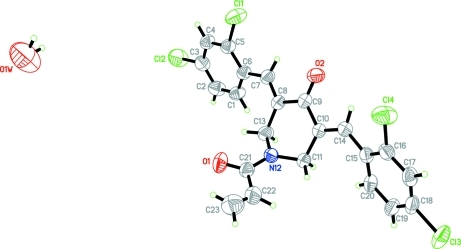
The asymmetric unit of the title compound, showing 30% probability displacement ellipsoids and the atom-numbering scheme.

**Fig. 2.**
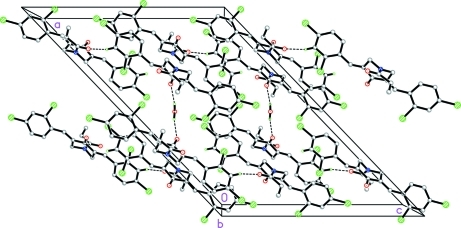
The crystal packing of the title compound (I) with hydrogen bonds shown as dashed lines. H atoms not involved in the intermolecular interactions have been omitted for clarity.

## supplementary crystallographic information

### Comment

The α,β-unsaturated carbonyl moiety is present in a large number of natural and synthetic products which are products of the Claisen-Schmidt condensation reaction. They exhibit wide variety of biological activities such as cytotoxicity (Oh *et al.*, 2006), antitumor (El-Subbagh *et al.*, 2000) and antimicrobial (Husain *et al.*, 2006) properties. Furthermore, it has been shown that the conjugated system plays a fundamental role in determining the bioactivity, due to its ability to act as a Michael acceptor for the addition of protein functional groups (Favier *et al.*, 2005). The title compound (I), is a new piperidin-4-one derivative.

The asymmetric unit of the title compound consists of  a (3*E*,5*E*)-1-acryloyl-3,5-bis(2,4-dichlorobenzylidene) piperidin-4-one molecule and a half-molecule of water (the O atom of the water molecule lies on a twofold axis), as shown in Fig. 1. The dihedral angle between the two terminal phenyl (C1–C6:C15–C20) rings is 8.84 (11)°.  The piperidine (N12/C8–C11/C13) ring adopts an envelope conformation [puckering parameters: Q = 0.508 (3) Å, θ = 122.4 (3)° and φ = 182.1 (4)°; (Cremer & Pople, 1975)] with atoms C11 and C13 deviating by 0.233 (2) and 0.217 (3) Å  from the least-squares plane defined by the remaining atoms (N12/C8–C10) in the ring.

In the crystal structure, (Fig. 2), the molecules are connected by intermolecular C4—H4A···O1 hydrogen bonds forming one-dimensional supramolecular chains along the *c*-axis.  Furthermore, adjacent chains are inter-connected by water molecules via O1W—H1W1···O2 hydrogen bonds.

### Experimental

3,5-bis(2,4-dichlorobenzylidene)piperidin-4-one was synthesized by the method described by Dimmock *et al.*, (2000). Briefly, the title compound (I) was prepared by dropwise addition of acryloyl chloride solution (7.24 mmol) to stirring mixture of 3,5-bis(2,4-dichlorobenzylidene) piperidin-4-one (4.82 mmol) and acetone (10 ml) in presence of weak base at room temperature. After completion of the reaction (through TLC monitoring), the mixture was poured into ice. The precipitate was filtered and washed with water. The pure solid was then recrystallized from ethanol to afford the title compound as yellow crystals.

### Refinement

Atoms H23A and H23B  were located from a difference Fourier map and refined freely. The remaining H atoms were positioned geometrically [O–H = 1.0501 Å ] and were refined using a riding model, with *U*_iso_(H) = 1.2 or 1.5 *U*_eq_(C).

### Crystal data

**Table:**

2C_22_H_15_Cl_4_NO_2_·H_2_O	*F*(000) = 1944
*M**_r_* = 952.32	*D*_x_ = 1.513 Mg m^−^^3^
Monoclinic, *C*2/*c*	Mo *K*α radiation, λ = 0.71073 Å
Hall symbol: -C 2yc	Cell parameters from 4558 reflections
*a* = 27.0296 (12) Å	θ = 3.0–23.7°
*b* = 11.3031 (5) Å	µ = 0.59 mm^−^^1^
*c* = 18.9580 (14) Å	*T* = 296 K
β = 133.807 (2)°	Plate, yellow
*V* = 4180.0 (4) Å^3^	0.41 × 0.22 × 0.09 mm
*Z* = 4	

### Data collection

**Table:**

Bruker SMART APEXII CCD area-detector diffractometer	6084 independent reflections
Radiation source: fine-focus sealed tube	3314 reflections with *I* > 2σ(*I*)
graphite	*R*_int_ = 0.035
φ and ω scans	θ_max_ = 30.1°, θ_min_ = 2.1°
Absorption correction: multi-scan (*SADABS*; Bruker, 2009)	*h* = −37→37
*T*_min_ = 0.794, *T*_max_ = 0.947	*k* = −15→15
22264 measured reflections	*l* = −26→26

### Refinement

**Table:**

Refinement on *F*^2^	Primary atom site location: structure-invariant direct methods
Least-squares matrix: full	Secondary atom site location: difference Fourier map
*R*[*F*^2^ > 2σ(*F*^2^)] = 0.050	Hydrogen site location: inferred from neighbouring sites
*wR*(*F*^2^) = 0.140	H atoms treated by a mixture of independent and constrained refinement
*S* = 1.04	*w* = 1/[σ^2^(*F*_o_^2^) + (0.0565*P*)^2^ + 1.3835*P*] where *P* = (*F*_o_^2^ + 2*F*_c_^2^)/3
6084 reflections	(Δ/σ)_max_ < 0.001
273 parameters	Δρ_max_ = 0.32 e Å^−^^3^
0 restraints	Δρ_min_ = −0.30 e Å^−^^3^

### Special details

**Table:**

Geometry. All s.u.'s (except the s.u. in the dihedral angle between two l.s. planes) are estimated using the full covariance matrix. The cell s.u.'s are taken into account individually in the estimation of s.u.'s in distances, angles and torsion angles; correlations between s.u.'s in cell parameters are only used when they are defined by crystal symmetry. An approximate (isotropic) treatment of cell s.u.'s is used for estimating s.u.'s involving l.s. planes.
Refinement. Refinement of F^2^ against ALL reflections. The weighted R-factor wR and goodness of fit S are based on F^2^, conventional R-factors R are based on F, with F set to zero for negative F^2^. The threshold expression of F^2^ > 2σ(F^2^) is used only for calculating R-factors(gt) etc. and is not relevant to the choice of reflections for refinement. R-factors based on F^2^ are statistically about twice as large as those based on F, and R- factors based on ALL data will be even larger.

### Fractional atomic coordinates and isotropic or equivalent isotropic displacement parameters (Å^2^)

**Table:**

	*x*	*y*	*z*	*U*_iso_*/*U*_eq_	
Cl1	0.19792 (3)	0.76399 (7)	0.69001 (5)	0.0713 (2)	
Cl2	0.42591 (5)	0.54693 (7)	0.82870 (7)	0.0917 (3)	
Cl3	0.03129 (4)	0.56068 (7)	1.19385 (5)	0.0758 (2)	
Cl4	−0.03353 (4)	0.74207 (6)	0.87822 (6)	0.0793 (2)	
O1	0.18932 (9)	0.28214 (16)	0.88038 (12)	0.0674 (5)	
O1W	0.0000	0.0870 (4)	0.2500	0.1570 (17)	
H1W1	−0.0368	0.1548	0.2124	0.235*	
O2	0.13682 (9)	0.76238 (14)	0.86903 (13)	0.0622 (5)	
C1	0.33740 (12)	0.57044 (19)	0.92958 (17)	0.0525 (6)	
H1A	0.3468	0.5447	0.9845	0.063*	
C2	0.38244 (13)	0.5423 (2)	0.92100 (19)	0.0599 (6)	
H2A	0.4215	0.4978	0.9691	0.072*	
C3	0.36913 (13)	0.5808 (2)	0.84014 (19)	0.0553 (6)	
C4	0.31230 (12)	0.64792 (19)	0.76899 (17)	0.0520 (6)	
H4A	0.3040	0.6748	0.7152	0.062*	
C5	0.26793 (11)	0.67438 (18)	0.77940 (15)	0.0463 (5)	
C6	0.27789 (11)	0.63624 (18)	0.85892 (15)	0.0432 (5)	
C7	0.23003 (11)	0.66960 (19)	0.86785 (15)	0.0450 (5)	
H7A	0.2063	0.7399	0.8371	0.054*	
C8	0.21566 (11)	0.61329 (18)	0.91388 (14)	0.0428 (5)	
C9	0.16516 (11)	0.66934 (18)	0.91293 (15)	0.0450 (5)	
C10	0.15150 (11)	0.61138 (17)	0.96889 (14)	0.0415 (5)	
C11	0.18741 (13)	0.49604 (19)	1.02007 (17)	0.0514 (6)	
H11A	0.2303	0.5116	1.0866	0.062*	
H11B	0.1589	0.4479	1.0226	0.062*	
N12	0.20085 (11)	0.43276 (15)	0.96822 (15)	0.0519 (5)	
C13	0.24670 (13)	0.4960 (2)	0.96662 (18)	0.0544 (6)	
H13A	0.2556	0.4483	0.9339	0.065*	
H13B	0.2900	0.5100	1.0331	0.065*	
C14	0.10773 (11)	0.66486 (18)	0.96949 (15)	0.0435 (5)	
H14A	0.0858	0.7311	0.9294	0.052*	
C15	0.08921 (11)	0.63488 (17)	1.02373 (15)	0.0412 (5)	
C16	0.02599 (12)	0.66960 (18)	0.98959 (16)	0.0485 (5)	
C17	0.00789 (12)	0.64771 (19)	1.04118 (18)	0.0525 (6)	
H17A	−0.0346	0.6715	1.0165	0.063*	
C18	0.05393 (12)	0.5901 (2)	1.12973 (17)	0.0505 (6)	
C19	0.11714 (12)	0.5554 (2)	1.16688 (16)	0.0513 (6)	
H19A	0.1481	0.5171	1.2269	0.062*	
C20	0.13390 (11)	0.57787 (19)	1.11453 (15)	0.0462 (5)	
H20A	0.1767	0.5543	1.1404	0.055*	
C21	0.17589 (12)	0.3248 (2)	0.92493 (16)	0.0501 (6)	
C22	0.13274 (16)	0.2603 (2)	0.9335 (2)	0.0657 (7)	
H22A	0.1364	0.2797	0.9847	0.079*	
C23	0.09076 (18)	0.1794 (3)	0.8743 (2)	0.0836 (9)	
H23A	0.0609 (16)	0.130 (3)	0.877 (2)	0.100*	
H23B	0.0891 (17)	0.161 (3)	0.826 (2)	0.100*	

### Atomic displacement parameters (Å^2^)

**Table:**

	*U*^11^	*U*^22^	*U*^33^	*U*^12^	*U*^13^	*U*^23^
Cl1	0.0626 (4)	0.0989 (5)	0.0619 (4)	0.0183 (3)	0.0467 (3)	0.0281 (3)
Cl2	0.1113 (7)	0.0861 (5)	0.1412 (7)	0.0305 (4)	0.1113 (6)	0.0297 (5)
Cl3	0.0849 (5)	0.1020 (6)	0.0794 (4)	−0.0122 (4)	0.0715 (4)	−0.0143 (4)
Cl4	0.0618 (4)	0.0870 (5)	0.0905 (5)	0.0289 (4)	0.0533 (4)	0.0392 (4)
O1	0.0783 (13)	0.0780 (12)	0.0699 (11)	0.0054 (9)	0.0603 (11)	−0.0090 (9)
O1W	0.191 (5)	0.105 (3)	0.240 (5)	0.000	0.174 (4)	0.000
O2	0.0806 (12)	0.0583 (10)	0.0728 (11)	0.0209 (8)	0.0626 (10)	0.0227 (8)
C1	0.0539 (14)	0.0573 (13)	0.0553 (13)	−0.0014 (11)	0.0412 (12)	0.0075 (11)
C2	0.0550 (15)	0.0579 (14)	0.0759 (16)	0.0061 (11)	0.0487 (14)	0.0129 (12)
C3	0.0637 (16)	0.0483 (12)	0.0806 (16)	−0.0016 (11)	0.0600 (14)	0.0012 (11)
C4	0.0634 (15)	0.0525 (13)	0.0579 (13)	−0.0086 (11)	0.0486 (13)	−0.0030 (10)
C5	0.0488 (13)	0.0467 (12)	0.0504 (12)	−0.0060 (10)	0.0371 (11)	−0.0010 (9)
C6	0.0466 (12)	0.0445 (11)	0.0462 (11)	−0.0090 (9)	0.0350 (10)	−0.0035 (9)
C7	0.0480 (13)	0.0474 (12)	0.0449 (11)	−0.0027 (9)	0.0342 (10)	−0.0013 (9)
C8	0.0459 (12)	0.0464 (11)	0.0419 (10)	−0.0002 (9)	0.0325 (10)	−0.0002 (9)
C9	0.0515 (13)	0.0463 (12)	0.0438 (11)	0.0006 (10)	0.0355 (11)	−0.0005 (9)
C10	0.0484 (12)	0.0411 (10)	0.0425 (10)	0.0012 (9)	0.0344 (10)	−0.0005 (8)
C11	0.0719 (16)	0.0474 (12)	0.0601 (13)	0.0108 (11)	0.0551 (13)	0.0074 (10)
N12	0.0748 (13)	0.0409 (9)	0.0746 (12)	0.0072 (9)	0.0647 (12)	0.0057 (9)
C13	0.0651 (15)	0.0558 (13)	0.0671 (14)	0.0107 (11)	0.0551 (13)	0.0123 (11)
C14	0.0479 (12)	0.0405 (11)	0.0455 (11)	−0.0005 (9)	0.0336 (10)	−0.0002 (8)
C15	0.0440 (12)	0.0374 (10)	0.0497 (11)	−0.0017 (9)	0.0352 (10)	−0.0052 (9)
C16	0.0528 (14)	0.0399 (11)	0.0594 (13)	0.0044 (10)	0.0413 (12)	0.0018 (9)
C17	0.0520 (14)	0.0522 (13)	0.0685 (15)	−0.0018 (11)	0.0474 (13)	−0.0081 (11)
C18	0.0591 (15)	0.0528 (12)	0.0588 (13)	−0.0081 (11)	0.0480 (12)	−0.0135 (11)
C19	0.0584 (15)	0.0586 (14)	0.0477 (12)	0.0013 (11)	0.0407 (12)	−0.0025 (10)
C20	0.0441 (12)	0.0536 (12)	0.0473 (11)	0.0023 (10)	0.0340 (10)	−0.0051 (9)
C21	0.0583 (14)	0.0524 (13)	0.0517 (12)	0.0147 (11)	0.0427 (12)	0.0103 (10)
C22	0.088 (2)	0.0556 (14)	0.0835 (18)	−0.0039 (14)	0.0704 (17)	−0.0055 (13)
C23	0.083 (2)	0.086 (2)	0.083 (2)	−0.0061 (18)	0.058 (2)	0.0030 (18)

### Geometric parameters (Å, °)

**Table:**

Cl1—C5	1.743 (2)	C11—N12	1.451 (3)
Cl2—C3	1.735 (3)	C11—H11A	0.9700
Cl3—C18	1.730 (2)	C11—H11B	0.9700
Cl4—C16	1.736 (2)	N12—C21	1.360 (3)
O1—C21	1.226 (3)	N12—C13	1.449 (3)
O1W—H1W1	1.0501	C13—H13A	0.9700
O2—C9	1.226 (2)	C13—H13B	0.9700
C1—C2	1.373 (3)	C14—C15	1.461 (3)
C1—C6	1.397 (3)	C14—H14A	0.9300
C1—H1A	0.9300	C15—C20	1.400 (3)
C2—C3	1.380 (3)	C15—C16	1.401 (3)
C2—H2A	0.9300	C16—C17	1.385 (3)
C3—C4	1.373 (3)	C17—C18	1.378 (3)
C4—C5	1.376 (3)	C17—H17A	0.9300
C4—H4A	0.9300	C18—C19	1.380 (3)
C5—C6	1.403 (3)	C19—C20	1.370 (3)
C6—C7	1.466 (3)	C19—H19A	0.9300
C7—C8	1.337 (3)	C20—H20A	0.9300
C7—H7A	0.9300	C21—C22	1.477 (4)
C8—C9	1.494 (3)	C22—C23	1.273 (4)
C8—C13	1.516 (3)	C22—H22A	0.9300
C9—C10	1.494 (3)	C23—H23A	1.01 (3)
C10—C14	1.336 (3)	C23—H23B	0.90 (3)
C10—C11	1.510 (3)		
			
C2—C1—C6	122.4 (2)	C13—N12—C11	112.94 (19)
C2—C1—H1A	118.8	N12—C13—C8	110.76 (19)
C6—C1—H1A	118.8	N12—C13—H13A	109.5
C1—C2—C3	119.2 (2)	C8—C13—H13A	109.5
C1—C2—H2A	120.4	N12—C13—H13B	109.5
C3—C2—H2A	120.4	C8—C13—H13B	109.5
C4—C3—C2	121.3 (2)	H13A—C13—H13B	108.1
C4—C3—Cl2	118.93 (19)	C10—C14—C15	129.64 (19)
C2—C3—Cl2	119.8 (2)	C10—C14—H14A	115.2
C3—C4—C5	118.2 (2)	C15—C14—H14A	115.2
C3—C4—H4A	120.9	C20—C15—C16	115.8 (2)
C5—C4—H4A	120.9	C20—C15—C14	123.2 (2)
C4—C5—C6	123.3 (2)	C16—C15—C14	120.84 (19)
C4—C5—Cl1	117.01 (17)	C17—C16—C15	122.5 (2)
C6—C5—Cl1	119.65 (17)	C17—C16—Cl4	117.38 (18)
C1—C6—C5	115.5 (2)	C15—C16—Cl4	120.08 (17)
C1—C6—C7	123.27 (19)	C18—C17—C16	118.8 (2)
C5—C6—C7	121.13 (19)	C18—C17—H17A	120.6
C8—C7—C6	129.2 (2)	C16—C17—H17A	120.6
C8—C7—H7A	115.4	C17—C18—C19	120.7 (2)
C6—C7—H7A	115.4	C17—C18—Cl3	119.11 (19)
C7—C8—C9	117.79 (19)	C19—C18—Cl3	120.14 (18)
C7—C8—C13	124.7 (2)	C20—C19—C18	119.4 (2)
C9—C8—C13	117.56 (18)	C20—C19—H19A	120.3
O2—C9—C8	120.56 (19)	C18—C19—H19A	120.3
O2—C9—C10	120.8 (2)	C19—C20—C15	122.6 (2)
C8—C9—C10	118.57 (18)	C19—C20—H20A	118.7
C14—C10—C9	117.60 (18)	C15—C20—H20A	118.7
C14—C10—C11	124.36 (19)	O1—C21—N12	120.6 (2)
C9—C10—C11	118.04 (19)	O1—C21—C22	121.0 (2)
N12—C11—C10	109.96 (17)	N12—C21—C22	118.4 (2)
N12—C11—H11A	109.7	C23—C22—C21	123.1 (3)
C10—C11—H11A	109.7	C23—C22—H22A	118.4
N12—C11—H11B	109.7	C21—C22—H22A	118.4
C10—C11—H11B	109.7	C22—C23—H23A	127.5 (18)
H11A—C11—H11B	108.2	C22—C23—H23B	117 (2)
C21—N12—C13	120.28 (19)	H23A—C23—H23B	116 (3)
C21—N12—C11	126.8 (2)		
			
C6—C1—C2—C3	0.5 (4)	C10—C11—N12—C13	62.9 (2)
C1—C2—C3—C4	0.9 (4)	C21—N12—C13—C8	118.9 (2)
C1—C2—C3—Cl2	179.49 (18)	C11—N12—C13—C8	−61.9 (2)
C2—C3—C4—C5	−1.2 (3)	C7—C8—C13—N12	−153.1 (2)
Cl2—C3—C4—C5	−179.73 (17)	C9—C8—C13—N12	26.5 (3)
C3—C4—C5—C6	0.0 (3)	C9—C10—C14—C15	−174.22 (19)
C3—C4—C5—Cl1	177.85 (17)	C11—C10—C14—C15	6.4 (4)
C2—C1—C6—C5	−1.5 (3)	C10—C14—C15—C20	29.1 (3)
C2—C1—C6—C7	−178.5 (2)	C10—C14—C15—C16	−155.2 (2)
C4—C5—C6—C1	1.3 (3)	C20—C15—C16—C17	−0.9 (3)
Cl1—C5—C6—C1	−176.51 (16)	C14—C15—C16—C17	−176.90 (19)
C4—C5—C6—C7	178.3 (2)	C20—C15—C16—Cl4	179.54 (15)
Cl1—C5—C6—C7	0.5 (3)	C14—C15—C16—Cl4	3.6 (3)
C1—C6—C7—C8	−29.5 (3)	C15—C16—C17—C18	0.3 (3)
C5—C6—C7—C8	153.7 (2)	Cl4—C16—C17—C18	179.86 (16)
C6—C7—C8—C9	178.76 (19)	C16—C17—C18—C19	0.4 (3)
C6—C7—C8—C13	−1.6 (4)	C16—C17—C18—Cl3	−179.14 (16)
C7—C8—C9—O2	1.8 (3)	C17—C18—C19—C20	−0.5 (3)
C13—C8—C9—O2	−177.8 (2)	Cl3—C18—C19—C20	179.05 (17)
C7—C8—C9—C10	−176.47 (19)	C18—C19—C20—C15	−0.2 (3)
C13—C8—C9—C10	3.9 (3)	C16—C15—C20—C19	0.9 (3)
O2—C9—C10—C14	−0.3 (3)	C14—C15—C20—C19	176.7 (2)
C8—C9—C10—C14	178.02 (19)	C13—N12—C21—O1	−3.3 (3)
O2—C9—C10—C11	179.2 (2)	C11—N12—C21—O1	177.6 (2)
C8—C9—C10—C11	−2.5 (3)	C13—N12—C21—C22	176.3 (2)
C14—C10—C11—N12	150.5 (2)	C11—N12—C21—C22	−2.8 (3)
C9—C10—C11—N12	−28.9 (3)	O1—C21—C22—C23	−22.3 (4)
C10—C11—N12—C21	−117.9 (2)	N12—C21—C22—C23	158.1 (3)

### Hydrogen-bond geometry (Å, °)

**Table:**

*D*—H···*A*	*D*—H	H···*A*	*D*···*A*	*D*—H···*A*
O1W—H1W1···O2^i^	1.05	2.19	3.180 (3)	157
C4—H4A···O1^ii^	0.93	2.29	3.186 (3)	162

Symmetry codes: (i) −*x*, −*y*+1, −*z*+1; (ii) −*x*+1/2, *y*+1/2, −*z*+3/2.
